# Supplementary material for: Genetic evidence that Nkx2.2 acts primarily downstream of Neurog3 in pancreatic endocrine lineage development
Source: eLife. 2017 Jan 10;6:e20010. doi: 10.7554/eLife.20010 (PMC5224921; doi:10.7554/eLife.20010)
Supplement: Figure 3—source data 2. — DOI: http://dx.doi.org/10.7554/eLife.20010.010 [file elife-20010-fig3-data2.pptx]

## Slide 1
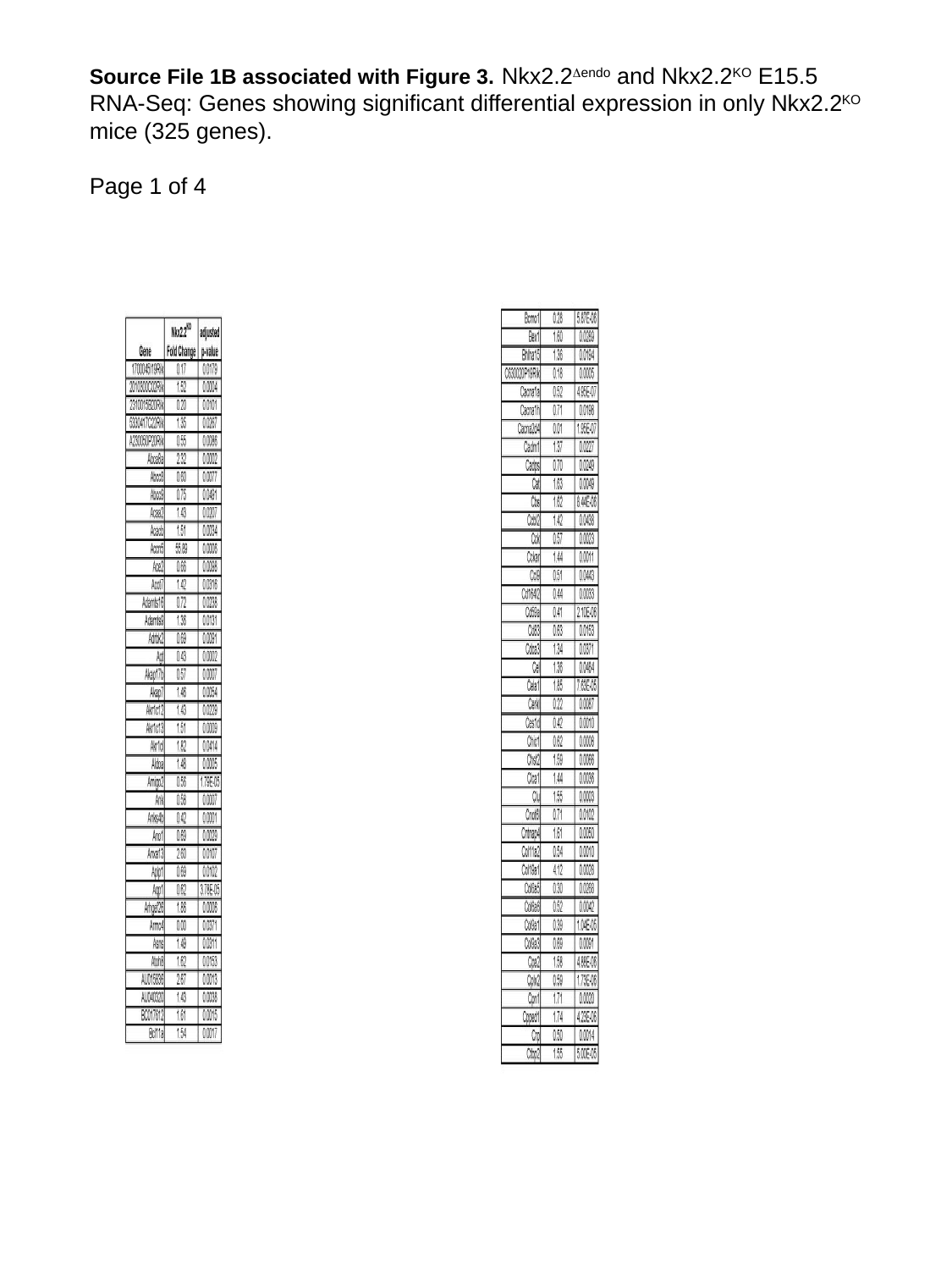

Source File 1B associated with Figure 3. Nkx2.2Dendo and Nkx2.2KO E15.5 RNA-Seq: Genes showing significant differential expression in only Nkx2.2KO mice (325 genes).
Page 1 of 4

## Slide 2
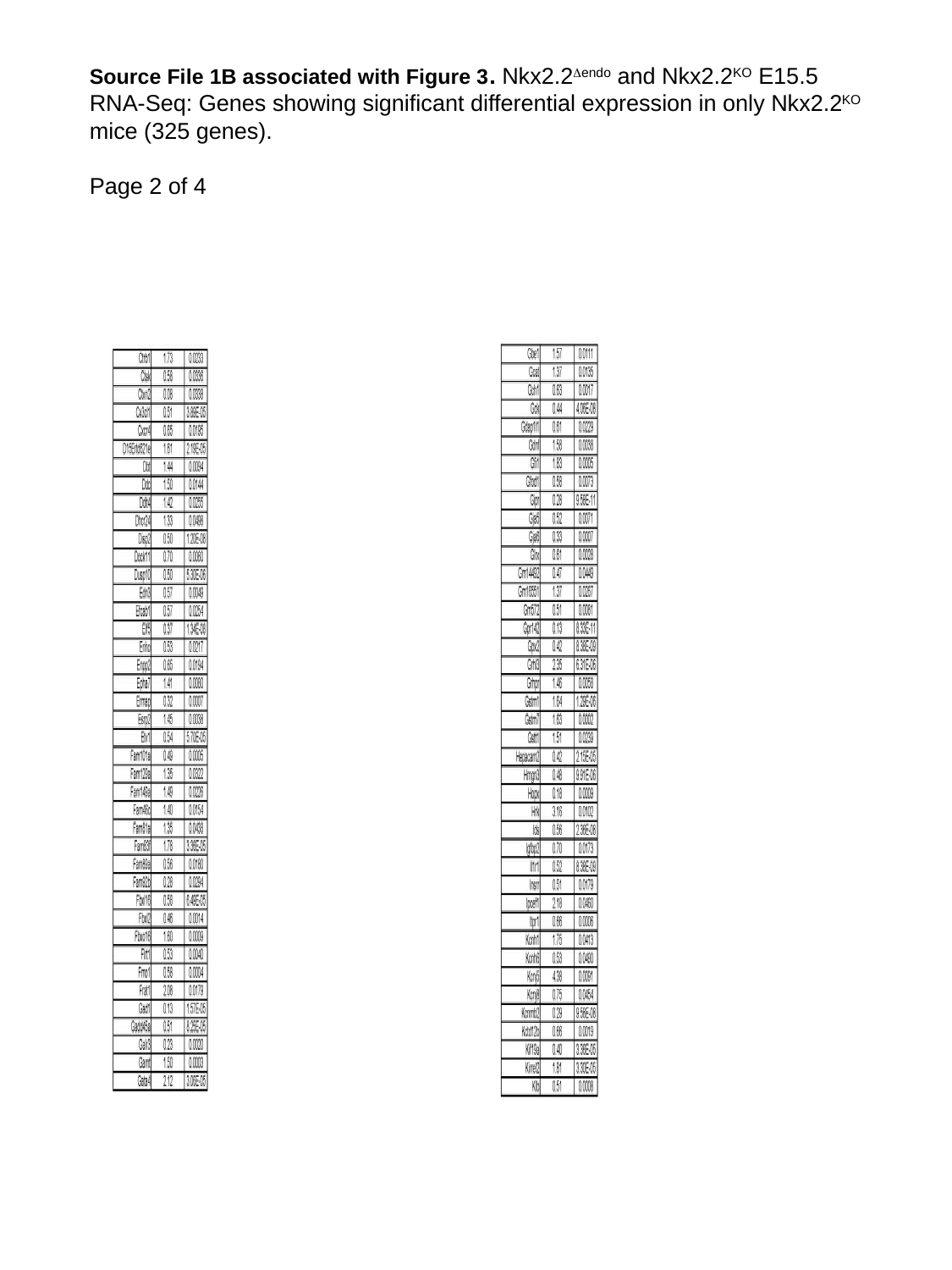

Source File 1B associated with Figure 3. Nkx2.2Dendo and Nkx2.2KO E15.5 RNA-Seq: Genes showing significant differential expression in only Nkx2.2KO mice (325 genes).
Page 2 of 4

## Slide 3
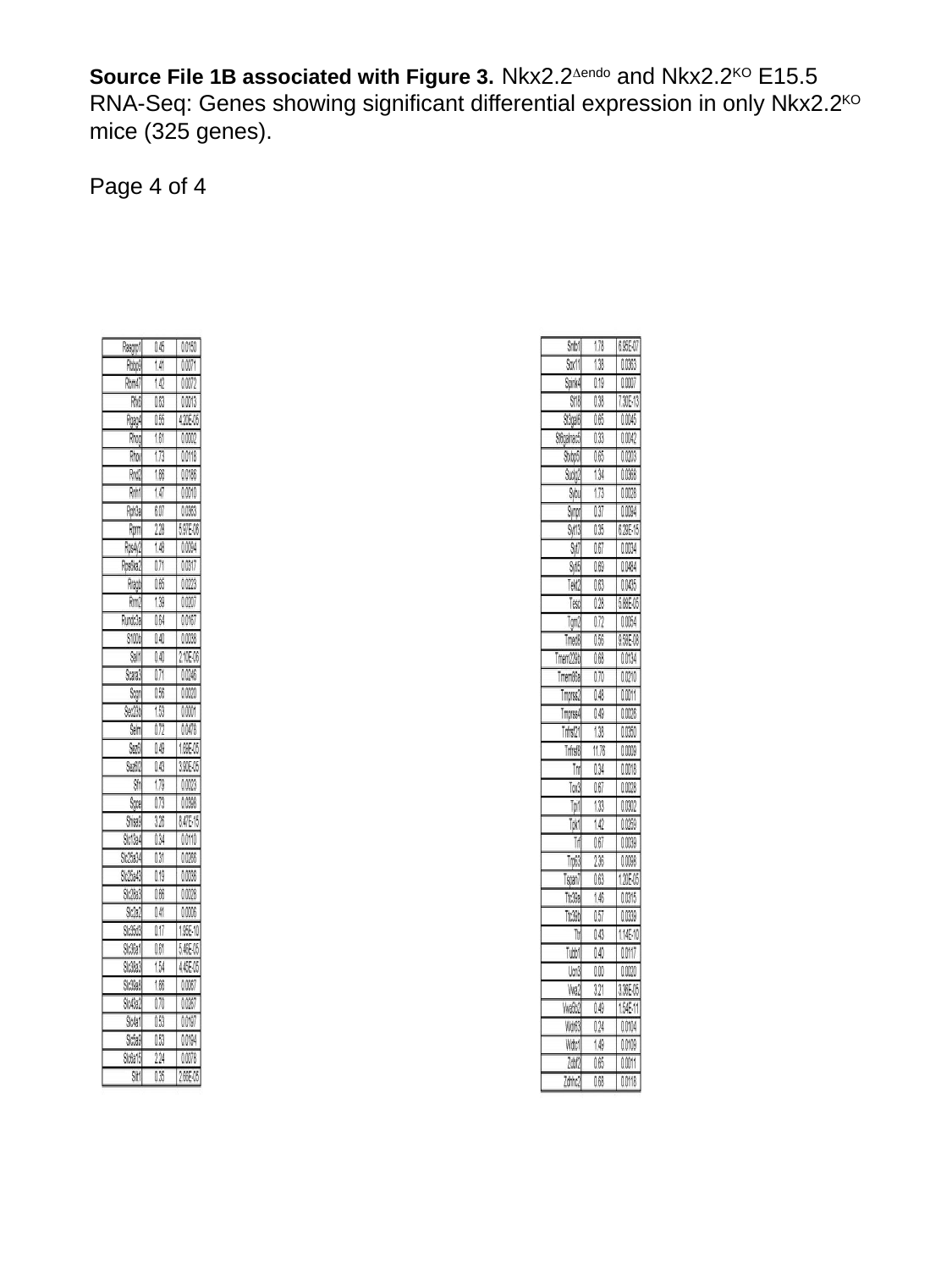

Source File 1B associated with Figure 3. Nkx2.2Dendo and Nkx2.2KO E15.5 RNA-Seq: Genes showing significant differential expression in only Nkx2.2KO mice (325 genes).
Page 4 of 4
