# Supplementary material for: Genetic evidence that Nkx2.2 acts primarily downstream of Neurog3 in pancreatic endocrine lineage development
Source: eLife. 2017 Jan 10;6:e20010. doi: 10.7554/eLife.20010 (PMC5224921; doi:10.7554/eLife.20010)
Supplement: Figure 3—source data 3. — DOI: http://dx.doi.org/10.7554/eLife.20010.011 [file elife-20010-fig3-data3.pptx]

## Slide 1
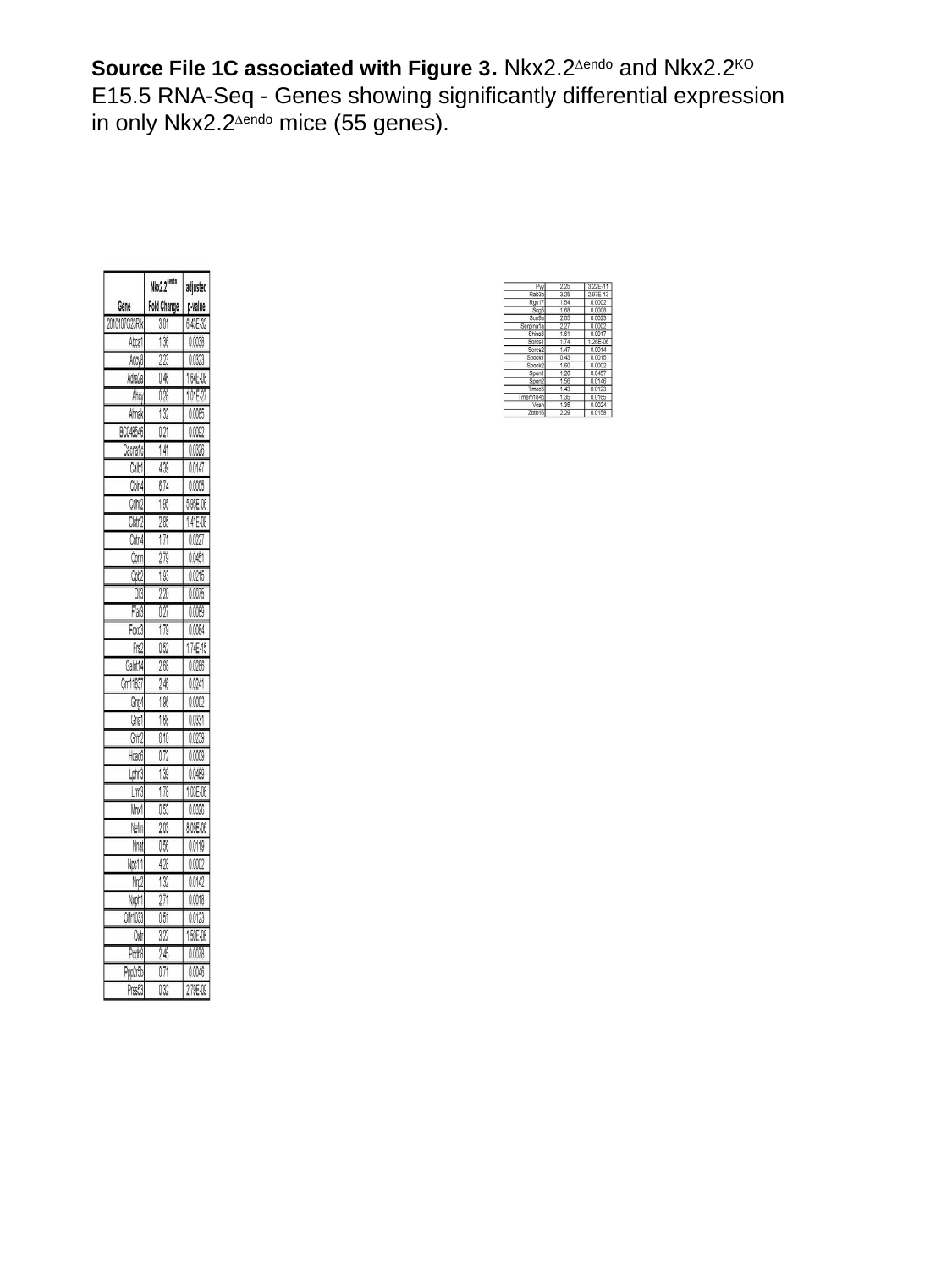

Source File 1C associated with Figure 3. Nkx2.2Dendo and Nkx2.2KO E15.5 RNA-Seq - Genes showing significantly differential expression in only Nkx2.2Dendo mice (55 genes).
