# Supplementary material for: Genetic evidence that Nkx2.2 acts primarily downstream of Neurog3 in pancreatic endocrine lineage development
Source: eLife. 2017 Jan 10;6:e20010. doi: 10.7554/eLife.20010 (PMC5224921; doi:10.7554/eLife.20010)
Supplement: Figure 4—source data 1. — DOI: http://dx.doi.org/10.7554/eLife.20010.014 [file elife-20010-fig4-data1.pptx]

## Slide 1
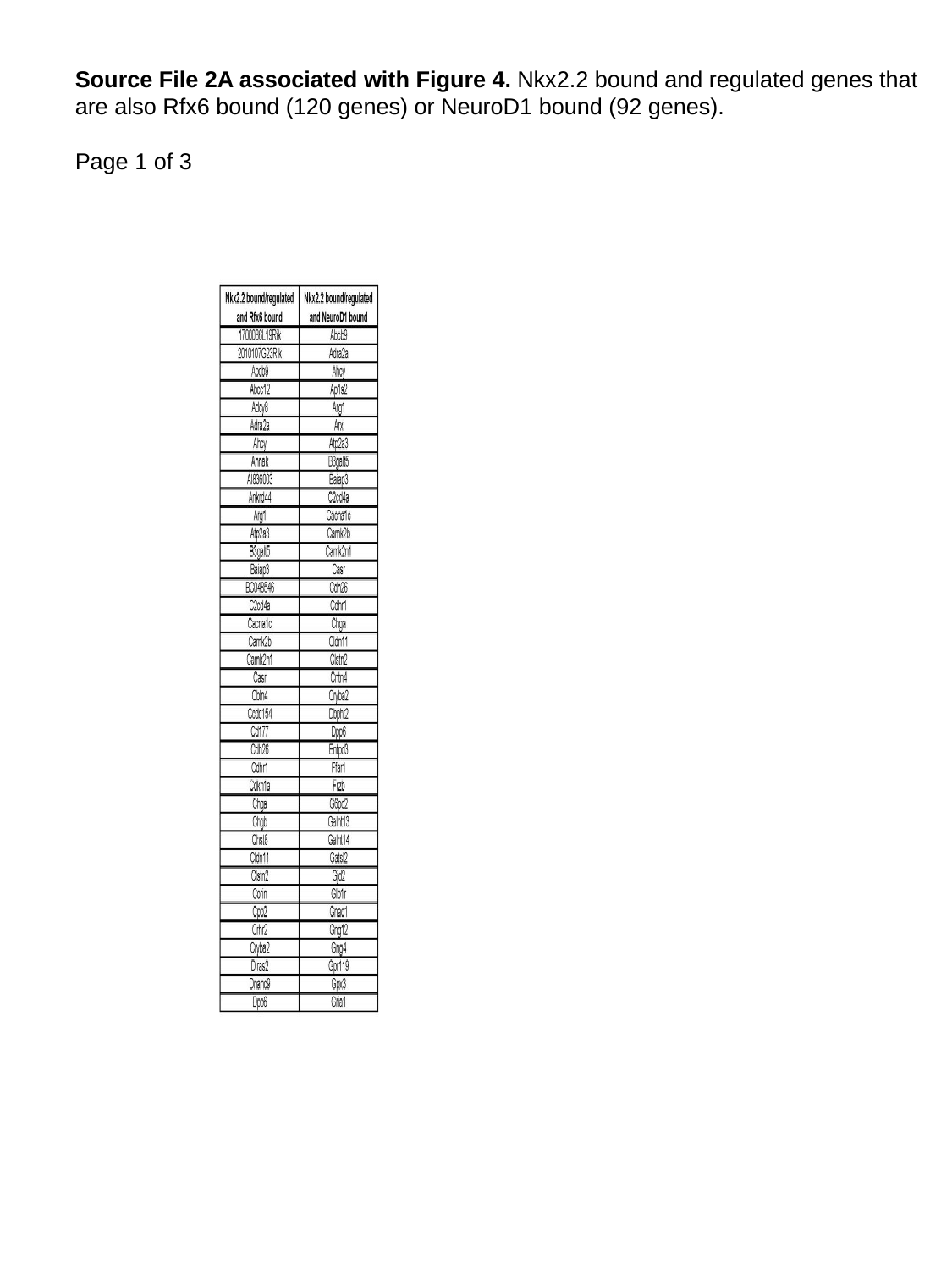

Source File 2A associated with Figure 4. Nkx2.2 bound and regulated genes that are also Rfx6 bound (120 genes) or NeuroD1 bound (92 genes).
Page 1 of 3

## Slide 2
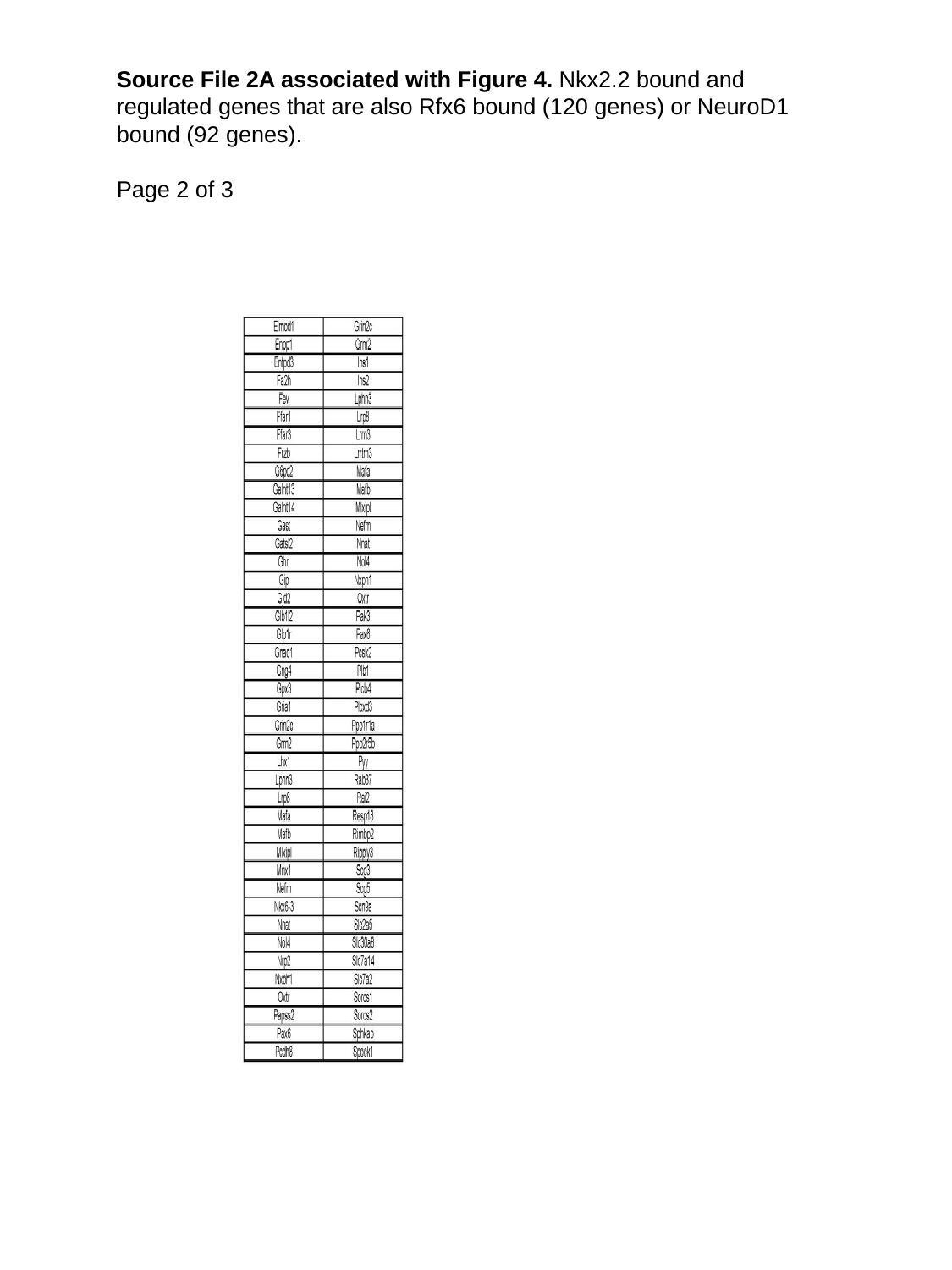

Source File 2A associated with Figure 4. Nkx2.2 bound and regulated genes that are also Rfx6 bound (120 genes) or NeuroD1 bound (92 genes).
Page 2 of 3

## Slide 3
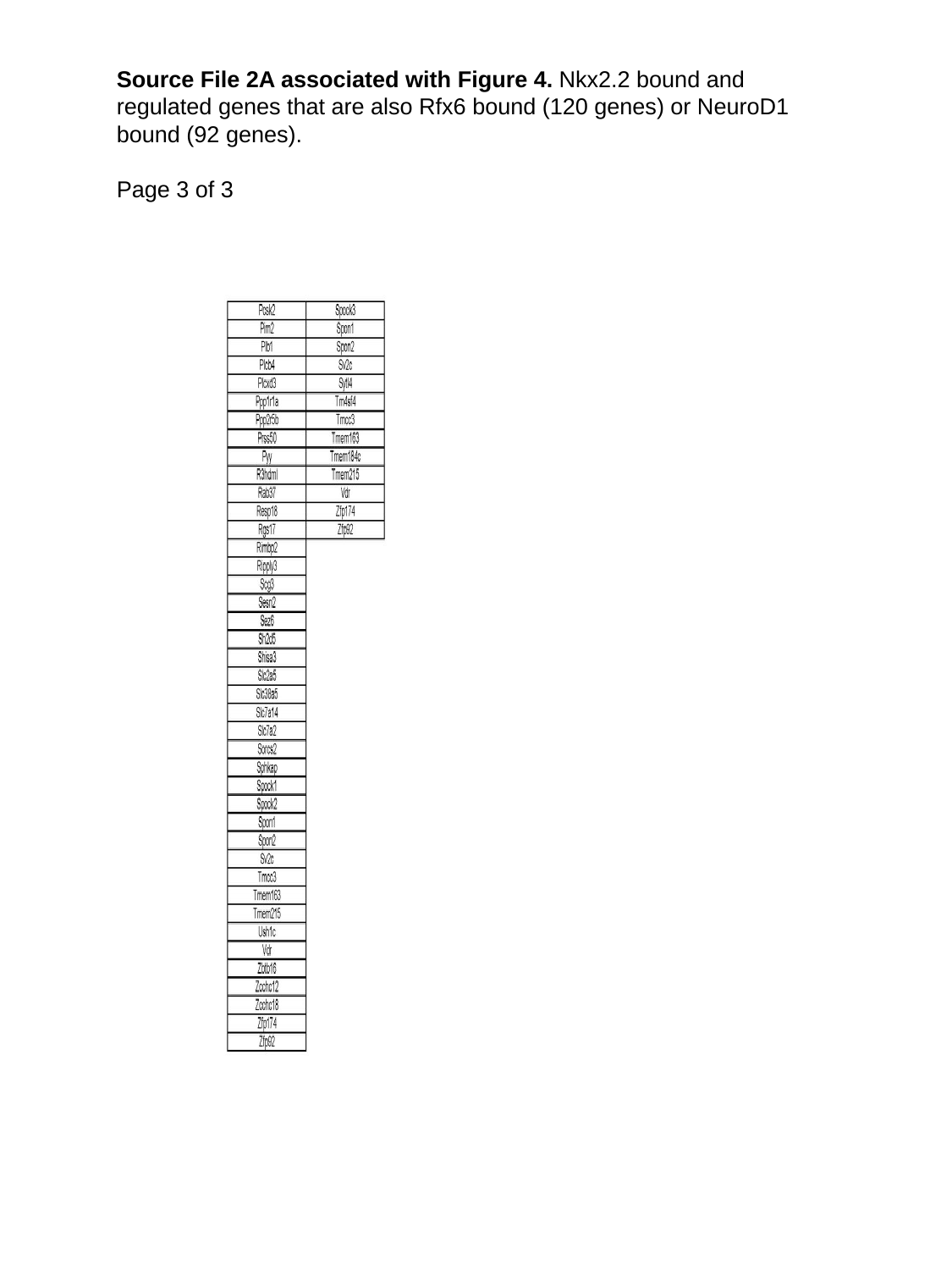

Source File 2A associated with Figure 4. Nkx2.2 bound and regulated genes that are also Rfx6 bound (120 genes) or NeuroD1 bound (92 genes).
Page 3 of 3
