# Supplementary material for: Genetic evidence that Nkx2.2 acts primarily downstream of Neurog3 in pancreatic endocrine lineage development
Source: eLife. 2017 Jan 10;6:e20010. doi: 10.7554/eLife.20010 (PMC5224921; doi:10.7554/eLife.20010)
Supplement: Figure 4—source data 2. — DOI: http://dx.doi.org/10.7554/eLife.20010.015 [file elife-20010-fig4-data2.pptx]

## Slide 1
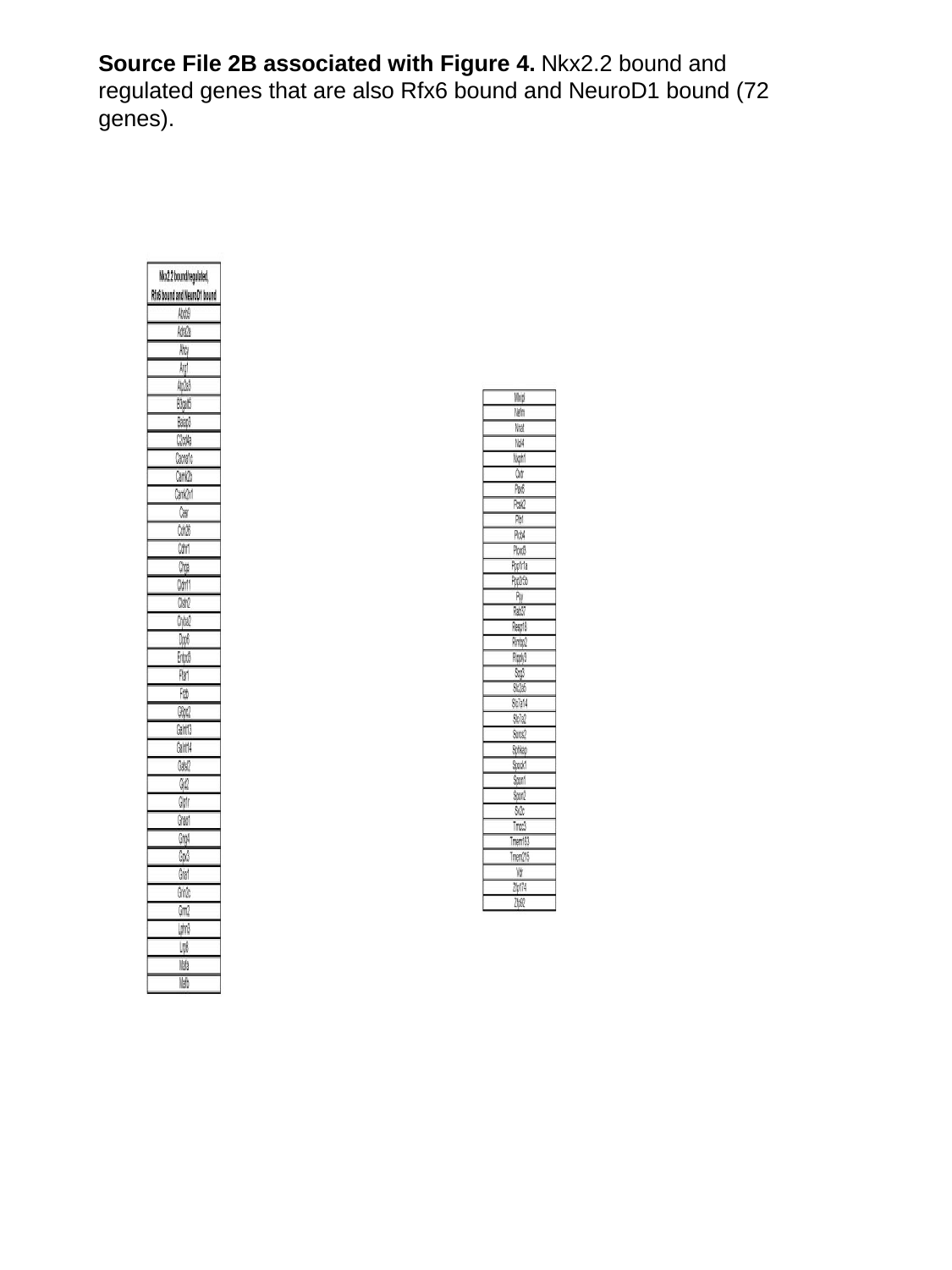

Source File 2B associated with Figure 4. Nkx2.2 bound and regulated genes that are also Rfx6 bound and NeuroD1 bound (72 genes).
